# Oral Potassium Supplementation Flicks the Renal K-Switch in Humans

**DOI:** 10.1016/j.ekir.2023.04.013

**Published:** 2023-04-23

**Authors:** Matthew A. Bailey

**Affiliations:** 1 Edinburgh Kidney Research Group; British Heart Foundation Center for Cardiovascular Science, The University of Edinburgh, Edinburgh, UK

See Clinical Research on Page 1201

High blood pressure is a major risk factor for cardiovascular, cerebrovascular, and renal disease. That dietary salt above recommended daily limits, typical for most people, directly contributes to hypertension and poor health outcomes is well established.^1^ The role of potassium intake, which conversely is often lower than recommended, is often overlooked. Indeed, there is a negative association between potassium intake and blood pressure, cardiovascular disease, and kidney disease. Many interventional studies targeting reduced sodium intake such as Dietary Approaches to Stopping Hypertension diets, and approaches that substitute regular table salt (100% NaCl) with “low salt” (75% NaCl, 25% KCl) concomitantly increase dietary potassium content.^1^ A meta-analysis of randomized controlled trials reported that oral potassium supplementation was associated with a mean systolic blood pressure reduction of approximately 3 mm Hg,^2^ similar to the reduction achieved by pharmacologic monotherapy. How increased potassium intake reduces blood pressure in humans is not well understood. One possibility is that high potassium intake improves the kidneys’ ability to excrete salt, thereby improving extracellular fluid-volume homeostasis.

Most of our body’s approximately 3.5 kg of potassium is stored within cells. Tight regulation of potassium concentration in extracellular fluid (normally 3.5–5.5 mmol/l) is important for maintaining the resting membrane potential of excitable cells. Perturbations outside this range, presenting as hypokalemia or hyperkalemia, are life-threatening.^3^ Oral potassium supplements, long recognized for their diuretic potential,^4^ can increase potassium to the top of the physiological range. Experiments in rodents have shown that such an increase in plasma potassium concentration is sufficient to inhibit the renal sodium-chloride cotransporter (NCC, encoded by *scl12a3*), expressed in the apical membrane of the distal convoluted tubule cell. NCC normally reabsorbs approximately 10% of the filtered sodium load. The transporter is activated by phosphorylation of serine and threonine residues in the N-terminal domain. Hyperphosphorylation of the transporter, as occurs in Gordon’s syndrome (Pseudohypoaldosteronism type II), causes hypertension. Dephosphorylation deactivates NCC and pharmacologic inhibition of the transporter by thiazide diuretics lowers blood pressure. Experiments giving an oral potassium supplement to mice show that the transporter is dephosphorylated, that is, deactivated.^5^ Using the isolated perfused mouse kidney or *ex vivo* tissue slices, this deactivating effect was found to be most sensitive as extracellular potassium increased from 4 mmol/l to 5 mmol/l, that is toward the top of the physiological range.^6^ Overall, these experiments suggest that oral potassium supplementation causes a natriuresis because of a thiazide-like effect, inhibiting tubular sodium transport.

The paper by Wu and colleagues, published in this issue of *KI Reports*,^7^ documents the first randomized, placebo-controlled crossover trial to test whether oral potassium supplementation directly regulates the NCC in humans as it does in rodents. Of course, it is not possible to fully-replicate the experimental studies in which NCC, both total and phosphorylated (i.e., activated) forms, is quantified by Western blotting in homogenates of kidney tissue. Instead, the investigators cleverly exploit the fact that renal tubule cells continuously shed extracellular vesicles containing nucleic acids and proteins reflective of ongoing biologic processes within the given cell.^8^ Here, abundance of NCC was measured in urinary extracellular vesicles as a window to the activity of the transporter within the distal convoluted tubule.

Healthy adults with normal blood pressure and plasma potassium were provided with a meal plan to ensure a core dietary intake that was high in salt (target 4.5 g sodium/d) and low in potassium (target 2.3 g/d) that did not change throughout the study. Each participant was presented in random order with both oral KCl supplement (72 mmol/d) or a placebo for 5 days, with a 2-day washout in between the intervention periods. A complete collection was obtained from each subject across the final 24-hours of each phase, from which urinary extracellular vesicles were isolated.

The potassium intervention significantly increased urinary potassium excretion and elevated plasma potassium within the physiological range at which the effect on NCC activity is most pronounced.^6^ The main finding of the study was that oral potassium reduced the amount of NCC, both total and phosphorylated forms, found in urinary extracellular vesicles. This is consistent with a downregulation of NCC expression in the distal convoluted tubule following oral potassium supplementation. Notably, the reduced expression of a key sodium transport protein was not associated with natriuresis; instead, sodium excretion was reduced and blood pressure, measured by 24-hour ambulatory blood pressure monitoring, was not significantly changed by the potassium intervention.

This study is important because it validates a mechanistic physiological hypothesis formulated from experiments in cell models and in rodents. The study by Wu *et al.*^7^ provides compelling evidence that the renal “K-switch” operates in humans and that oral potassium supplements can reduce the activity of the thiazide-sensitive sodium transporter in the kidney. The relevance of this for long-term human health is not yet clear. It is perhaps not surprising that potassium supplementation did not reduce blood pressure: this was a short duration study performed in a healthy, normotensive cohort. Any effect on blood pressure would be anticipated to be small, and the study was not powered for blood pressure as an endpoint. Nevertheless, Wu and colleagues have provided translation of discoveries originating in fundamental biologic research and this is a key threshold-step on the path to realizing potential health benefit.

I, for one, intend to eat more bananas.

## Disclosure

MAB acknowledges research funding from the British Heart Foundation (FS/4yPhD/F/21/34166, RE/18/5/34216).
